# *C. tropicali*s promotes chemotherapy resistance in colon cancer through increasing lactate production to regulate the mismatch repair system

**DOI:** 10.7150/ijbs.59262

**Published:** 2021-07-02

**Authors:** Junxing Qu, Zhiheng Sun, Chen Peng, Daoqian Li, Wenyue Yan, Zhen Xu, Yayi Hou, Sunan Shen, Ping Chen, Tingting Wang

**Affiliations:** 1 The State Key Laboratory of Pharmaceutical Biotechnology, Division of Immunology, Medical School, Nanjing University, Nanjing, China, 210093.; 2 Jiangsu Key Laboratory of Molecular Medicine, Division of Immunology, Medical School, Nanjing University, Nanjing, China, 210093.; 3 Department of Oncology, Yancheng First Hospital, Affiliated Hospital of Nanjing University Medical School, The First People's Hospital of Yancheng, Yancheng, Jiangsu, China, 224001.

**Keywords:** *Candida. tropicalis*, chemoresistance, glycolysis, lactate, mismatch repair

## Abstract

Due to chemotherapeutic drug resistance, tumor recurrence is common in patients with colorectal cancer (CRC) and chemo-resistant patients are often accompanied by defects in the mismatch repair system (MMR). Our previous study has shown that *Candida tropicalis* (*C. tropicalis*) is closely related to the occurrence and development of colorectal cancer, but whether this conditional pathogenic fungus is involved in chemotherapy needs further investigation. Here we found that *C. tropicalis* promoted chemotherapy resistance of colon cancer to oxaliplatin. Compared with oxaliplatin-treated group, the expression of functional MMR proteins in tumors were decreased in *C.tropicalis/*oxaliplatin -treated group, while the glycolysis level of tumors was up-regulated and the production of lactate was significantly increased in* C.tropicalis/*oxaliplatin -treated group. Inhibiting lactate production significantly alleviated the chemoresistance and rescued the decreased expression of MMR caused by *C. tropicalis*. Furthermore, we found that lactate down-regulated the expression of MLH1 through the GPR81-cAMP-PKA-CREB axis. This study clarified that *C. tropicalis* promoted chemoresistance of colon cancer via producing lactate and inhibiting the expression of MLH1, which may provide novel ideas for improving CRC chemotherapy effect.

## Introduction

Colorectal cancer (CRC) is the third most common cancer and the third leading cause of death in the world 1, which threatens human health and life safety. Current cancer treatments include chemotherapy, surgical treatment and immunotherapy 2, 3. The cytotoxic drugs commonly used in the therapy of colon cancer are oxaliplatin, 5-FU and capecitabine 4. Oxaliplatin, which is a kind of platinum, can form DNA-platinum crosslinks by covalently binding DNA to block the G2 phase, thereby suppressing tumor cell proliferation 5. Although patients with colorectal cancer can respond to chemotherapy at the beginning, they eventually experience relapse due to drug resistance 4. Therefore, it is very important to elucidate the mechanism of chemotherapy resistance in CRC patients.

Numerous underlying mechanisms conferring chemotherapy resistance have been elaborated in the past decades. Studies have reported that overexpression of ATP-binding cassette (ABC) transporters to enhance the efflux of anticancer medicines or escape chemotherapy-induced apoptosis can promote CRC chemotherapy resistance 6. Furthermore, a recent study discovered that tumor cells can evade chemotherapy by down-regulating the function of the mismatch repair system (MMR) to accumulate genetic mutations 7. MMR plays a prominent role in the correction of replicative mismatches 8. It is thought to involve certain functional proteins hMLH1, hMSH2, and so on 9. Studies have proved that loss of MMR could impair the ability to detect DNA damage and produce more mutations in tumor cells, which makes it more possible for tumor cells to improve adaptive variability and develop resistance to chemotherapeutics 8, 10, 11. Clinically, loss of MMR is a significant mechanism of resistance to many important drugs.

Accumulating evidence suggests that the gut microbiota including bacteria, fungi and virus plays a vital role in the cancer context. Studies have confirmed the association of *Peptostreptococcus stomatis*, *Parvimonas micra* and *Solobacterium moorei* with CRC 12. Microbial pathogens have been detected inside human tumor tissues and shown to promote cancer progression and resistance to anti-tumor therapies 13. Studies have shown that *Fusobacterium* significantly increased and could promote chemotherapy resistance in the course of CRC while other studies found that *Bifidobacterium* enhanced the anti-tumor effects 4, 13. Studies have found that intratumoral accumulation of *Bifidobacterium* facilitated CD47-based immunotherapy via STING signaling, and that the diversity and abundance of *Bifidobacterium* influenced the therapeutic outcome of blockade of the PD-1/PD- L1 axis 13, 14. However, most studies of commensal microbes focused on bacteria, commensal fungi have been less reported. Our previous study proved that the burden of *C. tropicalis*, which is a kind of conditional pathogenic fungus, is significantly elevated in both CRC patients and colon cancer animal models. *C. tropicalis* aggravated CRC by inducing the differentiation of myeloid-derived suppressor cells (MDSCs) and inhibiting the function of T cells 15. However, the potential effect of commensal fungi on chemotherapy is not examined in human literature.

In this study, we tested whether *C. tropicalis* had an effect on chemotherapy in the development of CRC. We have found that *C. tropicalis* can facilitate the glycolysis of tumor cells to produce lactate, which then inhibit the function of MMR system and promote the chemotherapy resistance of CRC to oxaliplatin.

## Materials and methods

### Reagents and Antibodies

The *C. tropicalis* strain (W4162870) was kindly provided by Dr. Sarah L. Gaffen (University of Pittsburgh, PA). Oxaliplatin(S1224), Forskolin (S2449) and Sodium Oxamate (S6871) were purchased from Selleck Chemicals (Houston, Texas, USA). Lactate was purchased from Sigma (St. Louis, MO, USA). Antibody against MLH1 (#3515), MSH2 (#2017), Cleaved caspase3 (#9664), caspase9 (#9508), p-H2AX (Ser139, #9718), PFKFB3 (#13123), LDHA (#3582), PKM2 (#4053), PGAM1 (#12098), GPI (#57893), GLUT1(#12939), ALDOA (#8060), HK2(#2867), PKAC-α(#4782) and p-CREB (Ser133, #9198)were purchased from Cell Signaling Technology (Boston, MO, USA). Antibody against β-tublin (# 66240-1-Ig) was purchased from Proteintech (Wuhan, Hubei, P.R.C). Antibody against GPR81 (YN2554) was purchased from Immunoway (Plano, TX, USA). CCK8 Kit (CK04) was purchased from Dojindo Laboratories (Japan). Annexin V FITC/PI Apoptosis Kit (A211-01) was purchased from Vazyme Biotech (Nanjing, China). F4/80-APC (#123115), CD11b-FITC (#301330), Ly6G-PE (#127607), Ly6C-APC (#128016), Gr-1-FITC (#108405) were all from Biolegend (San Diego, CA, USA). Lactate Assay Kit I (#120001100P) and Glucose Assay Kit I (#120003100P) were purchased from Eton Bioscience (San Diego, USA). Cyclic AMP Complete ELISA Kit (ab133051) was purchased from abcam (Cambridge, England).

### Mice

For CRC xenograft experiments, four-week-old male BALB/c nude mice were purchased from Model Animal Research Center of Nanjing University and housed in laminar flow cabinets under specific pathogen-free conditions. Colorectal cancer cells (1 × 10^7^ SW480 cells) were injected subcutaneously into the right axilla of each mouse. Six days after subcutaneous inoculation, mice were randomly divided into different groups (n=6 each group). *C. tropicalis* was given by multipoint intratumoral injection, twice per week for three weeks. Oxaliplatin (10 mg/kg) and Sodium oxamate (500 mg/kg) were administered by intraperitoneal injection, twice per week for three weeks. The length and width of the tumors were measured every three days. After three weeks, all mice were sacrificed and subcutaneous tumors were collected and weighed. All animal procedures were performed in accordance with the “National Institutes of Health Guidelines for the Care and Use of Laboratory Animals” and were approved by the Institutional Animal Care and Use Committee of Nanjing University Medical School.

### Fungi Strain and Growth Conditions

The *Candida tropicalis* strain (W4162870) was kindly provided by Dr. Sarah L. Gaffen (University of Pittsburgh, PA). *C. tropicalis* was cultured overnight at 30 °C in liquid Sabouraud Dextrose Medium and shake cultivation at 220 rpm/min to expand.

### TUNEL Assay

Tumor cell apoptosis in the xenograft tumor tissues were detected by terminal deoxynucleotidyl transferase-mediated dUTP nick end labeling (TUNEL) technology according to standard procedures.

### Immunohistochemistry

Mice tumor sections were obtained from CRC xenograft models and formalin fixed, paraffin-embedded, and processed according to standard procedures. Sections were analyzed with the following antibodies: anti-MLH1 (1:100, Proteintech, Wuhan, China); anti-MSH2 (1:200, CST, USA) and PCNA (1:200, CST, USA).

### RNA Extraction and Quantitative PCR

Total RNA was extracted from CRC cells using TRIzol reagent, and reverse transcriptions were performed in a 20 µL mixture with1 pg-1 µg of total RNA according to the manufacturer's instructions (Vazyme company, China). The oligonucleotide primers used for Quantitative real-time PCR amplification are listed in Table 1. PCR amplification consisted of 45 cycles of denaturation at 95 °C for 2 min, annealing at 60 °C for 30s, and extension at 72 °C for 30s on an Applied Biosystem Viia 7 quantitative PCR system (Applied Biosystems, Foster City, CA). Quantitative real-time PCR was performed by using SYBR Green with an ABI Step One Plus system (Life Technologies). All reactions were run in triplicate. The gene expression levels were normalized to β-tublin.

### Western Blot

The protein samples were obtained from lysis buffer-treated tumor tissues (30mg tumor section added 300ul lysis buffer and homogenized with 3 steel beads) or cells. Lysates were put on ice for 20 min and then centrifuged at14,000 × g for 10 min. Subsequently, BCA kit (Beyotime Biotechnology) was used to detect the protein concentration and 30 µg of protein per lane was separated on 10% and 15% polyacrylamide gels and transferred on to polyvinylidene difluoride membranes (Millipore, Billerica, MA, USA). Membranes were blocked with 5% bovine serum albumin (BSA) in Tris-buffered saline containing 0.1% Tween 20, and then the membranes were incubated with specific antibodies. The values were normalized to the β-tublin intensity levels.

### Nuclear protein extraction

A nuclear protein extraction kit was purchased from Beyotime Biotechnology (Wuhan, China) and used according to the manufacturer's instructions.

### Cell culture

Human colorectal cancer cell line SW480 and HCT116 were obtained from the Type Culture Collection of the Chinese Academy of Sciences (Shanghai, China) and were cultured in RPMI-1640 medium (GIBCO, Carlsbad, CA) supplemented with 10% fetal bovine serum (FBS), 1% (100 U/mL penicillin and 100 ug/mL streptomycin) at 37 °C in a humidified 5% CO_2_ atmosphere. Cells were seeded onto different types of plates for further experiments when the cell density reached ~80%.

### Cell viability assay, apoptosis detection and migration experiment

To assess the effect of *C. tropicalis* and lactate on cell viability, a CCK-8 assay was used according to the manufacturer's instructions (Dojindo Laboratories, Japan). CRC cells were seeded onto 96-well plates at a concentration of ~5 × 10^4^ cells/well with 100 μL culture medium. Different concentrations of *C. tropicalis* and lactate were used to treat cells for different length of time. The 10 μL of CCK-8 solution was added to the cells at specific time points and cells were incubated for 2 hr at 37 °C.

Apoptosis was examined by flow cytometric analysis. An Annexin V FITC/PI double stain assay (Vazyme, China) was performed following the manufacturer's protocol. Cell migration was measured by wound healing assay. CRC cells were divided into plates and the head of the gun was used to line with a ruler next day, then the cells were washed gently with PBS for 3 times. The cells were cultured in serum-free medium and photographed in certain times.

### ELISA

The concentration of lactate and glucose in cell supernatant and mice serum were detected using the corresponding enzyme-linked immunosorbent assay (ELISA) kit according to the manufacturer's instructions (Eton Bioscience), and cyclic AMP complete in the cell was detected using the corresponding mouse enzyme-linked immunosorbent assay (ELISA) kit according to the manufacturer's instructions (abcam).

### Immunofluorescence

Cultured cells were seeded on glass coverslips in six-well plates. After three PBS washes, the samples were fixed for 10 min at room temperature with 4% paraformaldehyde. Fixed cells were rinsed with PBS and then incubated for 10 min at 4 °C with 0.2%Triton X-100. Following permeabilization, non-specific binding in the cells was blocked by 5% BSA in PBS for 1 h at room temperature. Cell samples were incubated with anti-p-H2AX, anti-PKA Cα, and anti-p-CREB primary antibodies at a1:200 dilution for 2 h at room temperature. Samples were further incubated with Alexa 647-conjugated secondary antibody at a 1:400 dilution for 1.5 h in the dark. After washed with PBS, the nuclei were stained by DAPI. Slides were visualized using a confocal laser scanning microscope (FV3000, Olympus).

### siRNA Transfection

siRNA was transfected according to the product instructions (Ruibo company, China). The concentration of siRNA used in the study was 50 nM. The GPR81 siRNA target sequence is CTGCTAGACTCTATTTCCT. The target sequence of non-coding (NC) siRNA was a random sequence with no biological effects.

### Flow cytometric analysis

All splenocytes were flushed out from spleen by 1× PBS and then passed through a 200-mesh sieve to obtain single cell suspension. For flow cytometric detection, the following anti-mouse antibodies (all from Biolegend, San Diego, CA, US) were used: F4/80-APC, CD11b-FITC, Ly6G-PE, Ly6C-APC, Gr-1-FITC. Cells were detected using a FACS Calibur flow cytometer (Becton Dickinson, Franklin Lakes, NJ). Data were analyzed using FlowJo software (Treestar, Inc., San Carlos, CA).

### Statistical Analysis

Statistical analysis was conducted in GraphPad Prism 7. A two-tailed Student's t-test was employed to analyze statistical significance between two groups. Data are shown as means ± SD. P < 0.05 was considered to be statistically significant.

## Results

### *C. tropicalis* promotes the chemotherapy resistance to oxaliplatin in colon cancer

**Figure 1:**
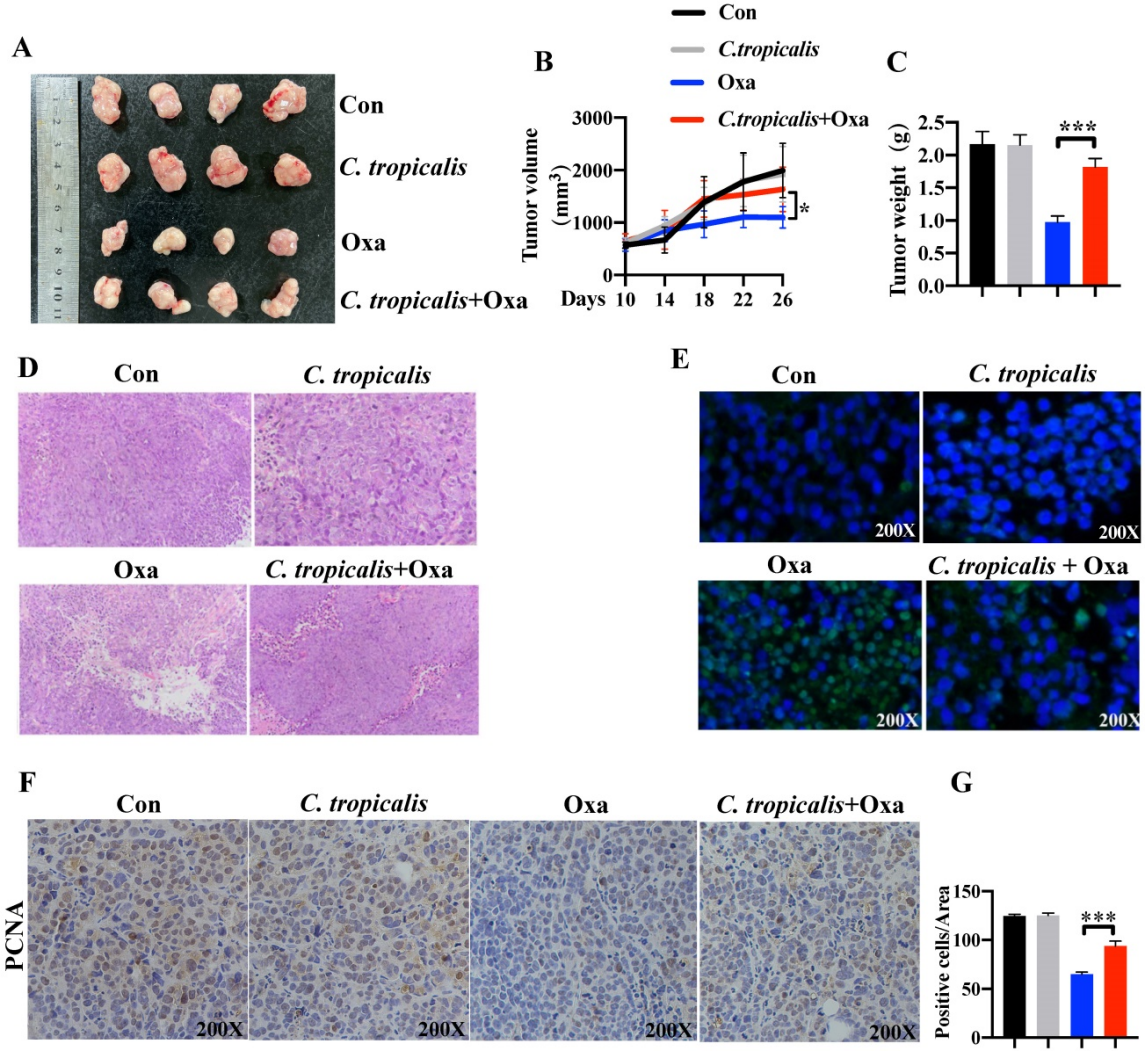
***C. tropicalis* promotes the chemotherapy resistance of colon cancer to oxaliplatin in mouse xenograft tumor model.** Mice were treated as described in Figure S1A. Tumors were acquired. (**A**) Representative data of tumors in mice under different conditions. (**B-C**) Statistical analysis of tumor volumes (B) and weights (C) in different groups, n=4/group. (**D**) Histological analysis of tumors was shown by hematoxylin and eosin staining. Tumors were microscopically analyzed. (**E**) TUNEL assays were performed to detect tumor cell apoptosis in tumor tissues. (**F and G**) Representative images of immunohistochemical staining of tumors in different groups for PCNA. Positive cells of PCNA were counted using Image-Pro Plus software 6.0. Data with error bars are represented as mean ± SD. Each panel is a representative experiment of at least three independent biological replicates. *p < 0.05, **p < 0.01 and ***p<0.001 as determined by unpaired Student's t test.

To explore the potential role of *C. tropicalis* on chemotherapy effect, oxaliplatin and *C. tropicalis* were injected into mice intraperitoneally and intratumorally in separately to establish the SW480 cells-induced xenograft CRC mouse model (Figure S1A). We found oxaliplatin treatment inhibited tumor growth, presenting as decreased tumor volume and tumor weight. *C. tropicalis* treatment significantly blocked the inhibitory effect of oxaliplatin (Figure 1A-C). However, *C. tropicalis* treated alone has no effect on tumor growth. Moreover, HE staining and analysis showed that tumors in oxaliplatin and *C. tropicalis* co-treatment mice had higher histological scores than those in oxaliplatin-treated mice (Figure 1D). TUNEL assay showed that *C. tropicalis* inhibited the tumor apoptosis caused by oxaliplatin (Figure 1E). In consistence with that, IHC staining showed more PCNA positive cells in tumors tissues from co-treatment mice than those from oxaliplatin-treated mice (Figure 1F-G). We also detected the proportion of immune cells in spleen of the tumor-bearing mice. As shown in Figure S1B, compared to oxaliplatin simply-treated mice, more proportion of macrophage, myeloid-derived suppressor cells (MDSCs) and G-MDSCs but lower frequency of M-MDSCs were found in co-treatment tumor-bearing mice.

**Figure 2:**
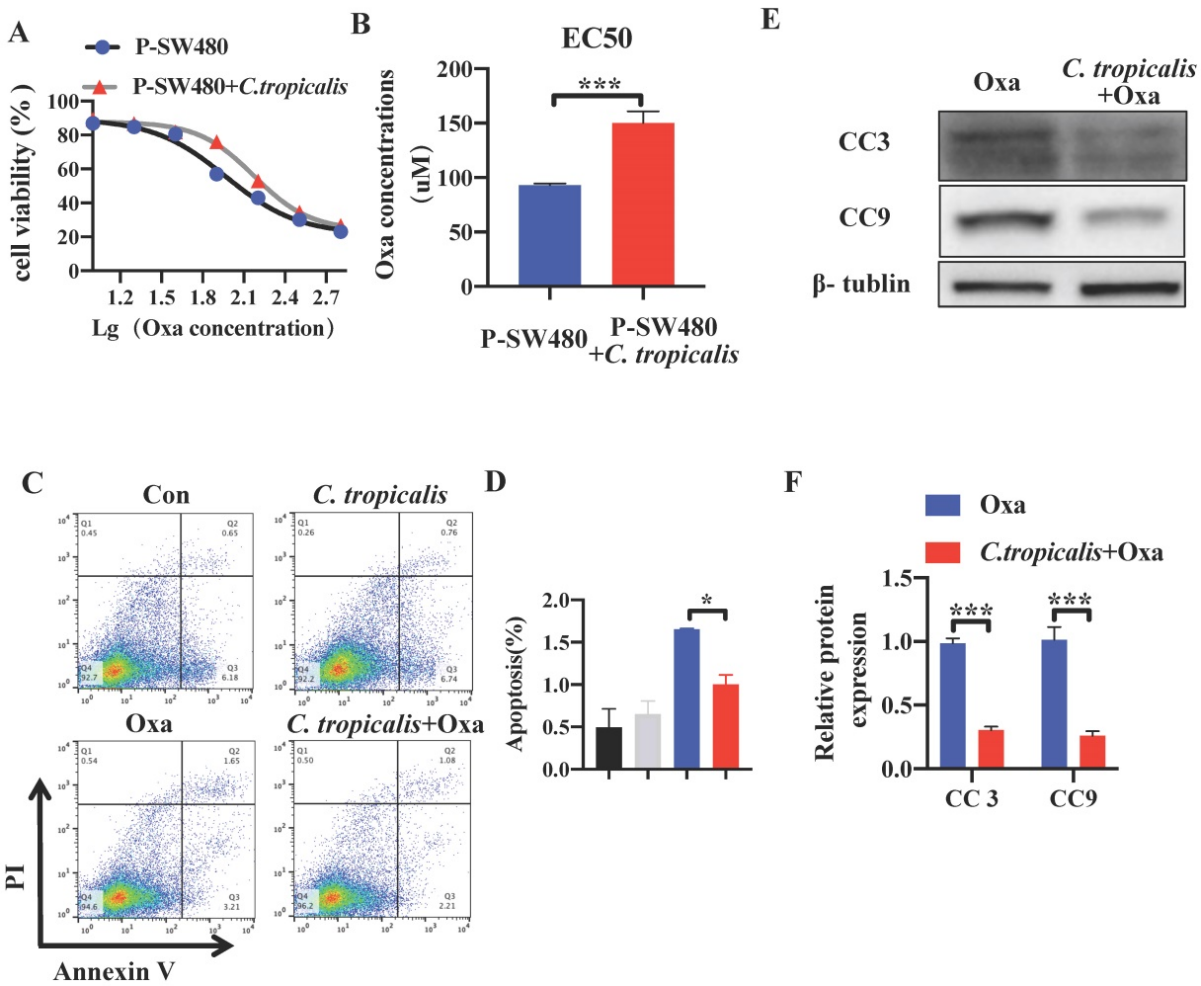
***C. tropicalis* promotes the chemotherapy resistance in SW480 cells.** SW480 cells were treated with oxaliplatin in the presence or absence of *C. tropicalis*. (**A**) Cell viability was detected by using CCK8 assay. (**B**) The EC50 value in different SW480 cells was calculated. (**C-D**) Proportion of apoptotic cells was detected by flow cytometry. (**E-F**) Cleaved caspases 3(CC3) and 9 (CC9) expression were detected by western blot. Data with error bars are represented as mean ± SD. Each panel is a representative experiment of at least three independent biological replicates. *p < 0.05, **p < 0.01 and ***p<0.001as determined by unpaired Student's t test.

The effect of *C. tropicalis* on chemotherapy resistance was further confirmed *in vitro*. Although *C. tropicalis* (MOI = 1) itself had no effect on proliferation, migration and apoptosis in SW480 cells (Figures S1C-F). We compared cell viability among the parental SW480 cells and the *C. tropicalis*-co-cultured parental SW480 cells in the presence of different concentrations of oxaliplatin (Oxa). We found that cell viability was decreased upon oxaliplatin treated (Figure 2A). The EC50 value of oxaliplatin was higher in *C. tropicalis*-cultured SW480 cells, compared with parental SW480 cells (Figure 2B). Then we examined the inhibitory role of *C. tropicalis* in oxaliplatin-induced apoptosis, presenting as decreased proportion of apoptotic cells (Figure 2C-D) and decreased protein expression of cleaved-caspase 3 (CC3) and cleaved-caspase 9 (CC9) (Figure 2E-F) upon *C. tropicalis* treatment. These results suggested that* C. tropicalis* promotes the chemotherapy resistance to oxaliplatin in both xenograft CRC mice and SW480 cancer cells.

### MMR is inhibited in *C. tropicalis*-induced CRC chemotherapy resistance

**Figure 3:**
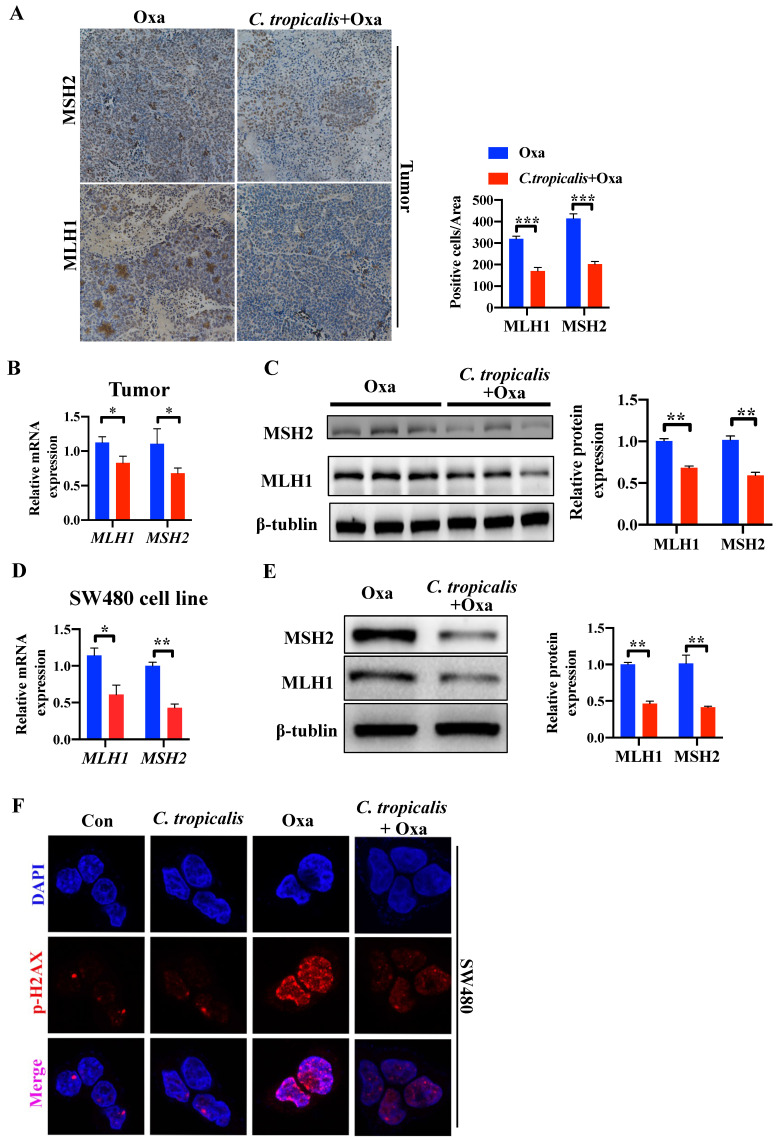
** MMR is inhibited upon *C. tropicalis* treatment.** (**A-C**) Mice were treated as described in Figure S1A. Tumors were acquired. Tumor tissues were stained for MLH1 and MSH2. The percentages of MLH1 and MSH2-positive tumor cells were quantified (A). mRNA expression of *MLH1* and *MSH2* in tumor tissues were detected by qPCR (B). Protein expression of MLH1 and MSH2 were detected by western blot (**C**). (**D-F**) SW480 cells were treated with oxaliplatin in the presence or absence of *C. tropicalis*. mRNA expression of *MLH1* and *MSH2* in cells were detected by qPCR (D). Protein levels of MLH1 and MSH2 were detected by western blot (E). p-H2AX expression were detected by immunofluorescence (F). Data with error bars are represented as mean ± SD. Each panel is a representative experiment of at least three independent biological replicates. Magnifications are 40× (scale bar, 0.05 mm) *p < 0.05, **p < 0.01 and ***p<0.001 as determined by unpaired Student's t test.

In our preliminary experiment, oxaliplatin treatment could significantly inhibit tumors in mice bearing SW480 tumor cells. However, oxaliplatin treatment had less inhibitory effect in mice bearing HCT116 tumor cells (Figure S2A-C). Several studies have shown that HCT116 is an MMR-deficient cell line while SW480 is an MMR-proficient cell line, and that loss of MMR leads to increased adaptive variability and chemoresistance in CRC 10, 16-18. Therefore, we detected the protein and mRNA expression of functional proteins in MMR. Compared with oxaliplatin-treated mice, *C. tropicalis* injection led to lower expression of MLH1 and MSH2 (Figure 3A-C). Similar result was found in SW480 cells (Figure 3D-E). Furthermore, quantification of phosphorylation of H2AX at Ser139, a common marker of DNA damage 10, suggested *C. tropicalis* inhibited oxaliplatin-induced DNA damage in SW480 cells (Figure 3F). These results suggest the expression of MMR functional proteins was inhibited upon *C. tropicalis* treatment.

### *C. tropicalis* promotes glycolysis and production of lactate in colon cancer

**Figure 4:**
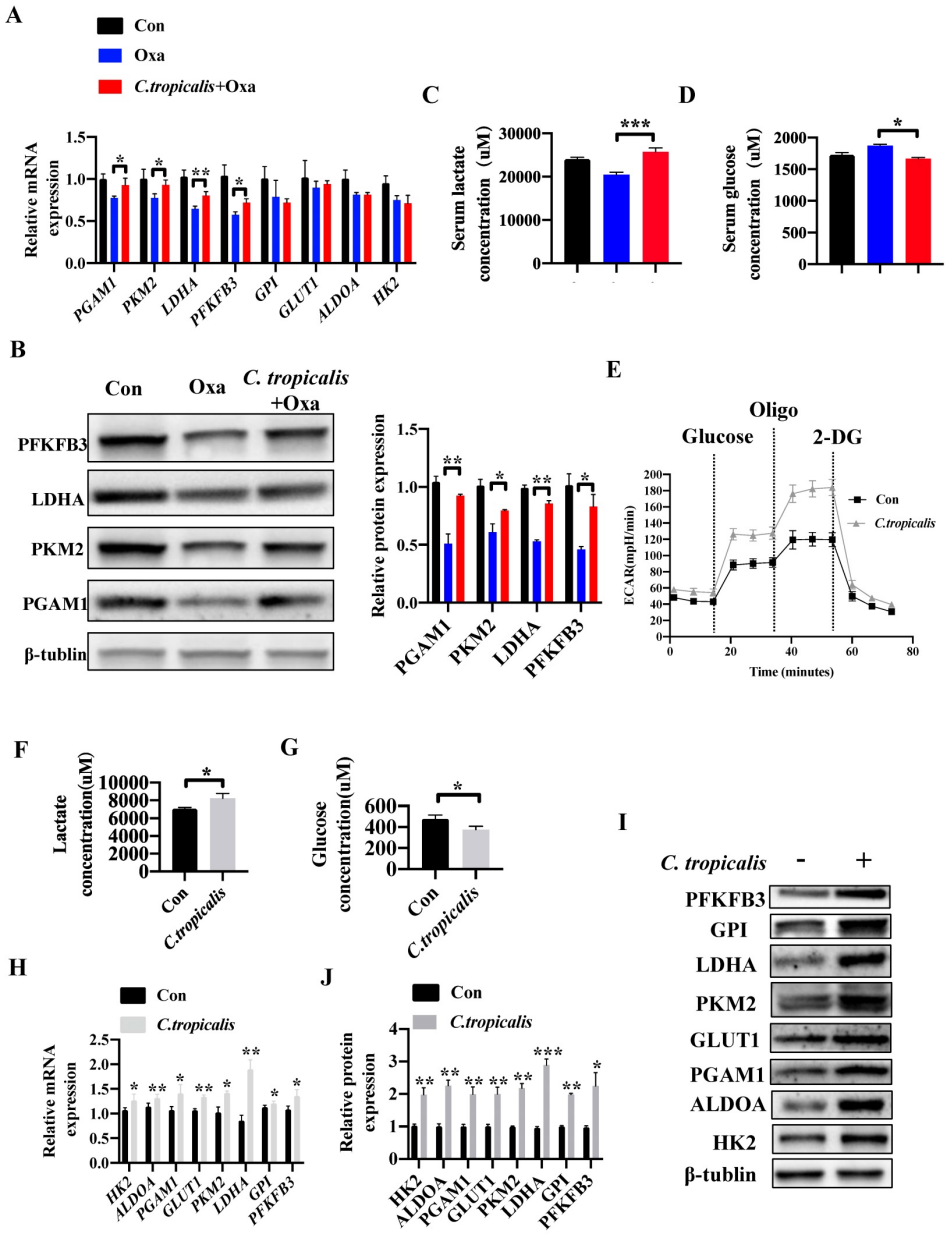
***C. tropicalis* promotes glycolysis and production of lactate in colon cancer.** (**A-D**) Mice were treated as described in Figure S1A. Tumors were acquired. mRNA expression of glycolysis-related enzymes in tumor tissues were detected using qPCR(A). Protein levels of glycolysis-related enzymes were detected using western blot (B). Lactate concentration in serum was detected by ELISA (C). Glucose concentration in serum was measured by ELISA (D). (**E-J**) SW480 cells were treated with or without *C. tropicalis*. The ECAR of SW480 cells was measured with a seahorse analyzer (E). Lactate level in cell supernatant was detected by ELISA (F). Glucose concentration in cell supernatant was detected by ELISA (G). mRNA expressions of key glycolysis-related enzymes were detected by qPCR (H). Protein expressions of key glycolysis-related enzymes were detected by western blot (I-J). Data with error bars are represented as mean ± SD. Each panel is a representative experiment of at least three independent biological replicates. *p < 0.05, **p < 0.01 and ***p<0.001 as determined by unpaired Student's t test.

Since “Warburg effect” is emerging as a symbol of cancer and a potential cause of chemotherapy resistance 19-24. We next explored whether *C. tropicalis* induced chemoresistance through regulating the level of glycolysis. Compared to oxaliplatin-treated mice, combination of *C. tropicalis* up-regulated the mRNA expression of pivotal glycolysis-related enzymes in tumor tissues, including *Pgam1*, *Pkm2*, *Ldha* and *Pfkfb3* (Figure 4A). Similar results were found in the protein levels of pivotal glycolytic enzymes (Figure 4B). Compared with oxaliplatin-treated mice, production of lactate in the serum was significantly increased in oxaliplatin and *C. tropicalis* co*-*treated mice while glucose concentration in the serum was decreased in co*-*treated mice (Figure 4C-D). We also detected the role of *C. tropicalis* on glycolysis pathway in colon cancer cells. Consistently, seahorse extracellular flux analysis showed that SW480 treated with *C. tropicalis* exhibited significantly higher glycolytic capacity than parental SW480 (Figure 4E). Lactate production and glucose consumption were also enhanced in SW480 cells upon *C. tropicalis* treatment (Figure 4F-G). *C. tropicalis* also up-regulated expression of vital glycolysis-related enzymes in SW480 cells in both mRNA and protein levels (Figure 4H-J). Therefore, these data suggest *C. tropicalis* promotes glycolysis and production of lactate in colon cancer.

### Inhibiting lactate attenuates *C. tropicalis-*induced chemoresistance to oxaliplatin

**Figure 5:**
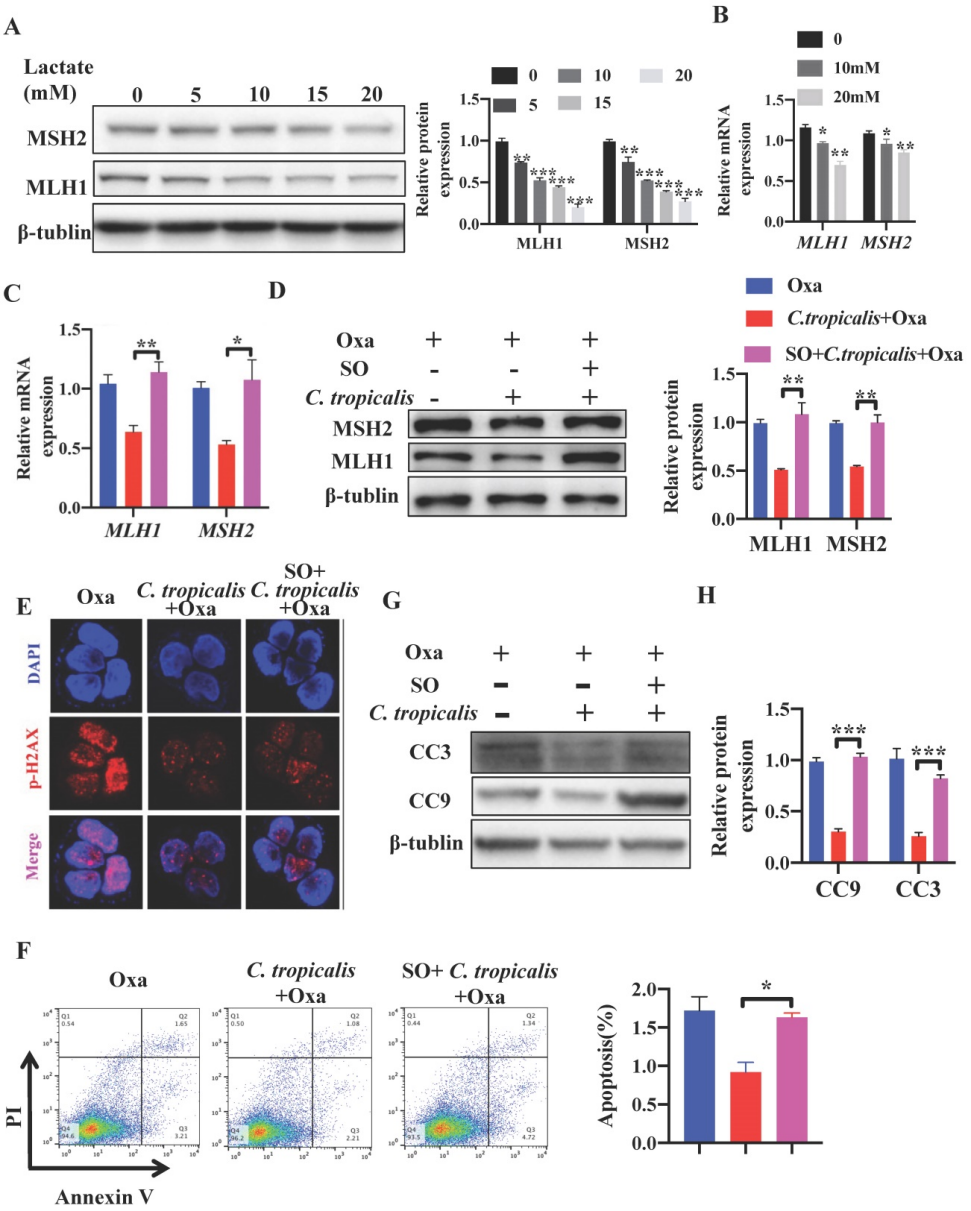
***C. tropicalis* promotes CRC chemotherapy resistance to oxaliplatin via producing lactate.** (**A-B**) SW480 cells were treated with lactate (5-20mM), protein and mRNA expression of MLH1 and MSH2 were detected by western blot and qPCR, respectively. (**C-H**) SW480 cells were stimulated with oxaliplatin in the presence or absence of *C. tropicalis* and Sodium Oxamate (SO). mRNA expressions of *MLH1* and *MSH2* in SW480 cells were measured by qPCR(C). (D) Protein expression of MLH1 and MSH2 were measured by western blot. p-H2AX expression was detected by immunofluorescence (E). Proportion of apoptotic cells was detected by flow cytometry (F). Protein levels of CC3 and CC9 were detected by western blot (G and H). Data with error bars are represented as mean ± SD. Each panel is a representative experiment of at least three independent biological replicates. *p < 0.05, **p < 0.01 and ***p<0.001 as determined by unpaired Student's t test.

Studies have reported that lactate is no longer just a metabolic waste, but a crucial signaling molecule that refer to the regulation of metabolic pathways 20, 23, 25-30. Next, we detected whether lactate contributed to the inhibition on MMR in *C. tropicalis*-induced chemoresistance. SW480 cells were treated with different concentrations of lactate. We found that 30 mM lactate significantly inhibited cell viability, therefore we used the concentration 0-20 mM in the following experiment (Figure S3A). Exogenous lactate triggered a dose-dependent decrease of MLH1 and MSH2, in both protein and mRNA levels (Figure 5A-B). To confirm whether lactate was critical in *C. tropicalis-*triggered chemoresistance, Sodium Oxamate (SO), a kind of LDHA inhibitor, was added in SW480 cells in the presence of oxaliplatin and *C. tropicalis.* We found that the decreased expression of MLH1 and MSH2 in SW480 cells treated with oxaliplatin and *C. tropicalis* was rescued due to the inhibition of LDHA (Figure 5C-D). Consistently, the decreased expression of p-H2AX was also rescued in the presence of SO (Figure 5E). Moreover, *C. tropicalis*-induced chemoresistance was also abolished by SO treatment in SW480 cells, presenting as increased proportion of apoptotic cells (Figure 5F) and increased expression of CC3 and CC9 (Figure 5G-H).

**Figure 6:**
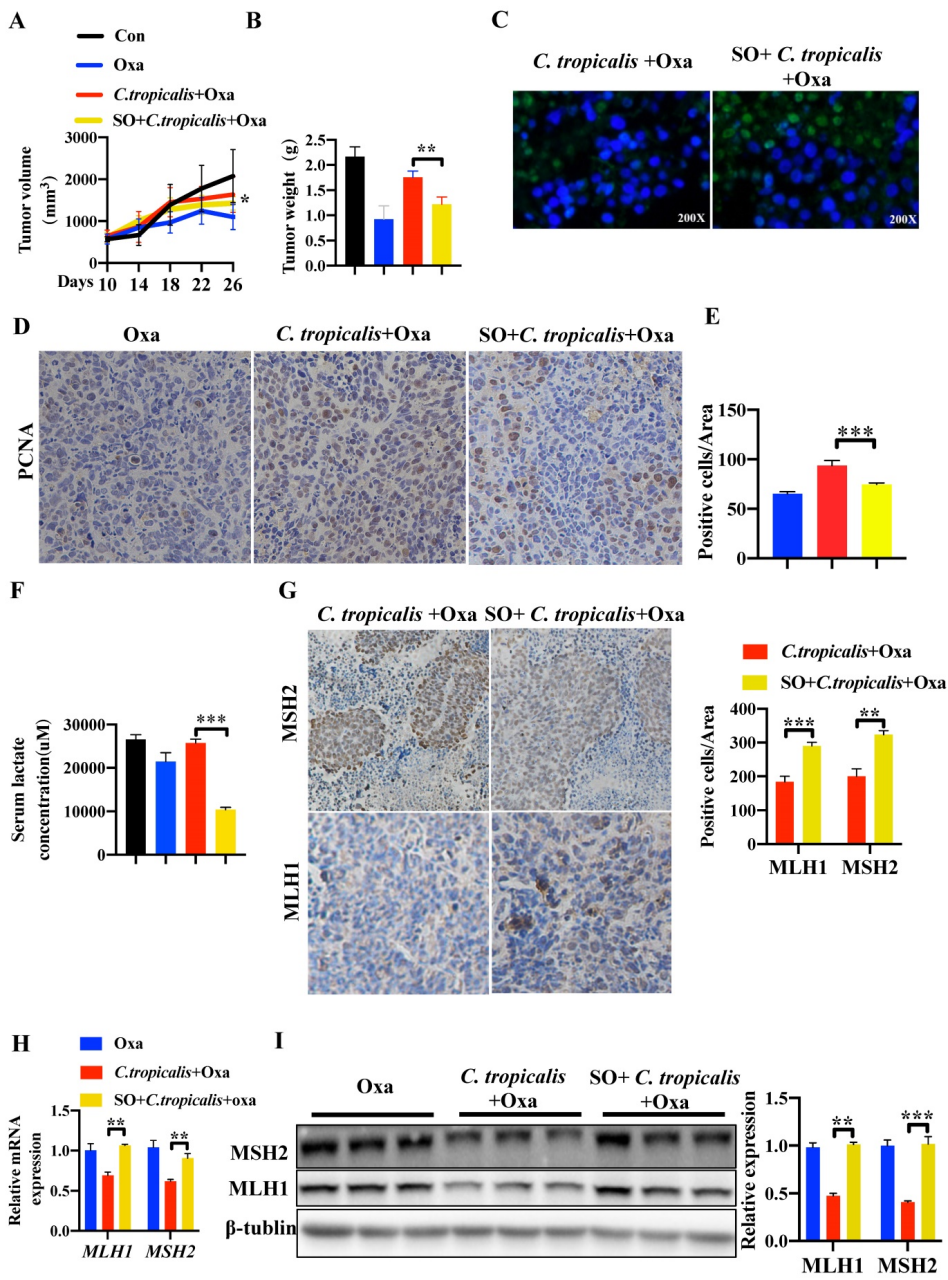
**Inhibiting lactate attenuates *C. tropicalis-*induced chemoresistance to oxaliplatin in CRC mice.** Mice were treated as described in Figure S1A and were injected SO intraperitoneally additionally. Tumors were acquired. (**A and B**) Statistical analysis of tumor volumes and tumor weights. (**C**) Apoptotic cells in tumor tissues were detected by TUNEL assays. (**D-E**) Representative images of IHC staining of tumors in different groups for PCNA. Positive cells of PCNA were counted using Image-Pro Plus software 6.0. (**F**) Lactate concentration in the serum was detected by ELISA. (**G**) Immunohistochemical staining of tumors and statistical analysis in different groups with anti-MLH1 and anti-MSH2 antibodies. (**H**) mRNA expression of *MLH1* and *MSH2* in tumor tissues was detected by qPCR. (**I**) Protein expression of MLH1 and MSH2 in tumor tissues was detected by Western blot. Data with error bars are represented as mean ± SD. Magnifications are 40× (scale bar, 0.05 mm). Each panel is a representative experiment of at least three independent biological replicates. *p < 0.05, **p < 0.01 and ***p<0.001 as determined by unpaired Student's t test.

We further confirmed this critical role of lactate in tumor-bearing mouse model. SO was intraperitoneal injected into mice during oxaliplatin and *C. tropicalis* treatment indicated as Figure S1A. We found that SO partly recovered the effect of oxaliplatin blocked by *C. tropicalis*, presenting as decreased tumor volume (Figure 6A) and tumor weight (Figure 6B). TUNEL assay showed that the inhibited tumor apoptosis caused by *C. tropicalis* was increased in presence of SO (Figure 6C). Consistently, IHC staining showed PCNA positive cells were increased in tumors tissues due to the inhibition of lactate (Figure 6D-E). We also confirmed that lactate production was indeed inhibited in the presence of SO (Figure 6F). Furthermore, IHC showed increased staining levels in the SO co-treated group compared with the oxaliplatin and *C. tropicalis*-treated group in tumor tissues, suggesting that the expression of MLH1 and MSH2 was rescued by SO (Figure 6G). Similar results were found in the mRNA and protein expression of MLH1 and MSH2 (Figure 6H-I). Our results demonstrated that inhibition of lactate can restore the down-regulated MMR functional proteins and attenuate chemotherapy resistance to oxaliplatin in CRC mice caused by *C. tropicalis*.

### Lactate reduces the expression of MLH1 via GPR81-cAMP-PKA-CREB axis

**Figure 7:**
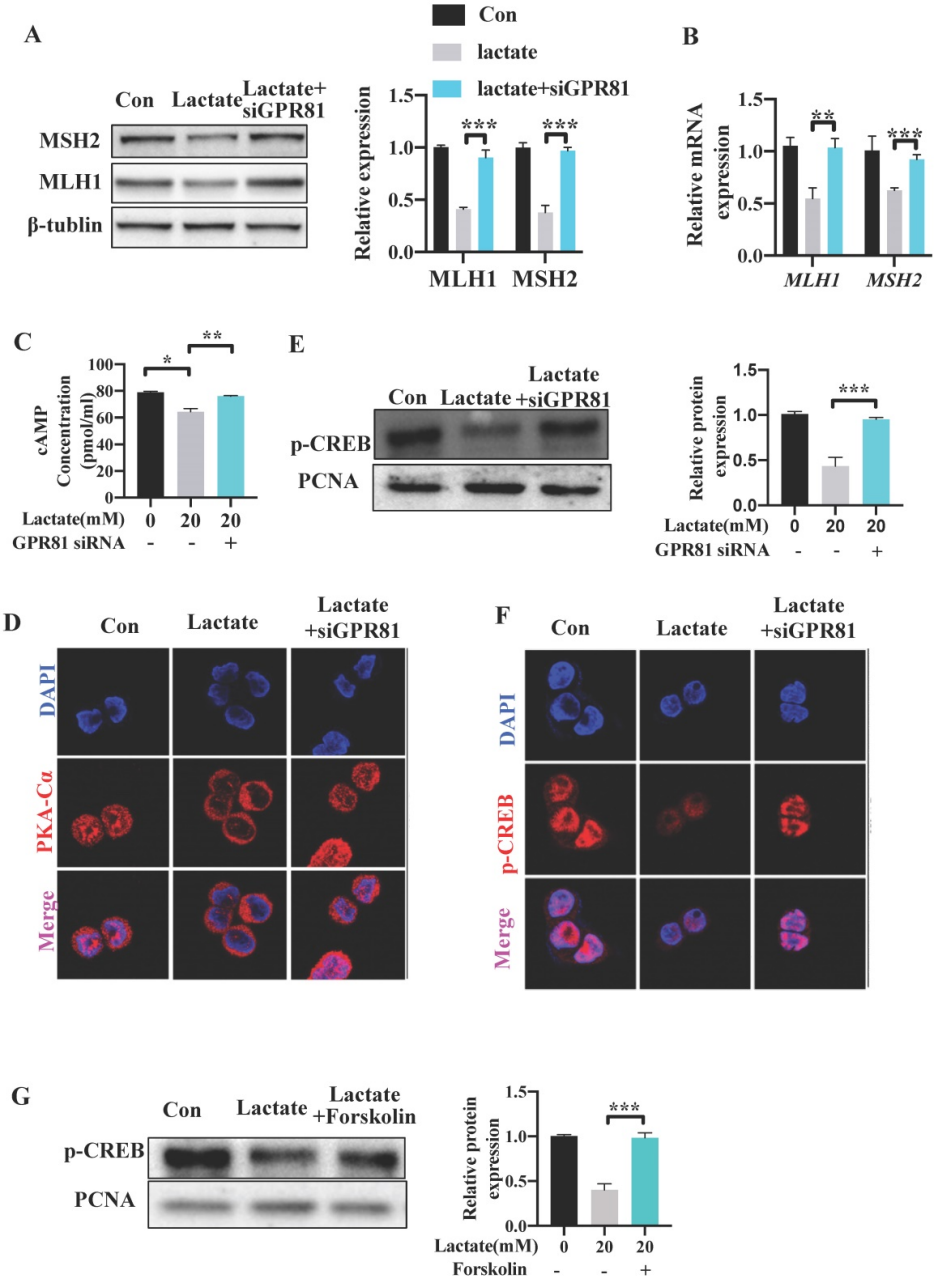
**Lactate reduces the expression of MLH1 via GPR81-cAMP-PKA-CREB axis.** SW480 cells were transfected with GPR81 siRNA and were then treated with or without lactate. (**A**) Protein expression of MLH1 and MSH2 was detected by Western blot. (**B**) mRNA expression of *MLH1* and *MSH2* was detected by qPCR. (**C**) Intracellular cAMP concentration was determined by ELISA. (D) Immunofluorescence indicated the nucleus translocation of PKACα cells. (**E-F**) Protein expression of p-CREB in nuclear was detected by western blot and immunofluorescence. (**G**) SW480 cells were treated with lactate in the presence or absence of Forskolin. Protein expression of p-CREB in nuclear was detected by western blot. Data with error bars are represented as mean ± SD. Each panel is a representative experiment of at least three independent biological replicates. *p < 0.05, **p < 0.01 and ***p <0.001 as determined by unpaired Student's t test.

Several studies have reported that lactate can bind and activate the G-protein coupled receptor GPR81 on the cell surface in an autocrine or paracrine manner 23, 31. To explore whether GPR81 mediates the inhibitory effect of lactate on the expression of MMR functional proteins, GPR81 was knocked down in SW480 cells by small interfering RNA siGPR81 (Figure S3B-C). Cells transfected with siGPR81 showed increased expression of MLH1 and MSH2 levels upon lactate stimulation (Figure 7A). Similar result was found in the mRNA expression of *MLH1* and *MSH2* (Figure 7B). Previous study has demonstrated that activation of GPR81 inhibited adenylate cyclase and results in a decreased cAMP level 32, 33. We found a decreased level of cAMP after lactate stimulation, which was recovered after transfecting with siGPR81 (Figure 7C). The activation and nuclear translocation of protein kinase A (PKA), which is the downstream of cAMP, was also inhibited by lactate, and was recovered by siGPR81 (Figure 7D). Next, we detected phosphorylation of cAMP response element-binding protein (p-CREB), which is a direct indicator of cAMP accumulation and PKA activation 23 and is also the upstream transcriptional factor of MLH1. Indeed, as a result of attenuation of cAMP concentration, p-CREB (Ser133) was decreased in response to lactate (Figure 7E-F). Meanwhile, siGPR81 blocked the inhibitory effect of lactate on PKA and p-CREB (Figure 7D-F). Cells were further treated with forskolin, a pharmacological activator of cAMP which can induce the production of cAMP. We found that increase of cAMP can enhance the nuclear translocation of PKA and the expression of p-CREB (Figure 7G, Figure S3D-E). These data showed that lactate regulated MMR protein expression via GPR81-cAMP-PKA-CREB pathway.

## Discussion

In this study, we find that *C. tropicalis* promotes CRC chemotherapy resistance and MMR functional proteins are down-regulated in these chemo-resistant tumor cells. *C. tropicalis* can markedly upregulate the glycolysis flux in CRC. As a product of glycolysis, lactate secretion is therefore significantly increased. Furthermore, lactate down-regulates the expression of MLH1 and MSH2 via GPR81-cAMP-PKA-CREB axis. Of note, *C. tropicalis*-induced CRC chemoresistance as well as the expression of MMR system is rescued after inhibition of lactate production. Thus, our study uncovers the role of *C. tropicalis* in the CRC chemotherapy resistance to oxaliplatin and the mechanisms by which lactate mediates the inhibition of MLH1 and MSH2. This consequence is the first to correlate conditional fungus with CRC chemoresistance. It plays an important role in solving the problem of relapse.

Current research has shown that the intestinal flora is related to CRC. Specific types of bacteria that promote tumorigenesis have been identified 34 and metagenomic analysis of the fecal microbiome has become a biomarker for colorectal cancer 12. Recent mouse studies have shown that the gut microbiota not only can modulate local immune responses but also affect chemotherapy 35, 36. *F. nucleatum* increases in patients with CRC recurrence and can promote colon cancer chemotherapy resistance by regulating modulating autophagy 4. Although bacteria are dominate in the microbe community, the role of fungi should not be underestimated. As for the commensal fungus, our previous studies have found *C. tropicalis* is specifically increased in *Dectin3*^-/-^colitis and *Card9*^-/-^ CRC mice 15, 37. However, no study has confirmed the role of commensal fungi in CRC chemoresistance until now. Here, we uncover the crucial role of *C. tropicalis* in the CRC chemotherapy resistance. By multipoint intratumoral injection of *C. tropicalis* into CRC xenograft mouse model, we provide compelling evidence that *C. tropicalis* activates glycolysis-related pathways to produce more lactate which down-regulates the MMR, and that *C. tropicalis* promotes CRC chemoresistance.

The Warburg effect is now emerging as a hallmark of cancer and an underlying cause of chemotherapy resistance 19-24. It is mainly manifested as enhanced glucose uptake, production of lactate 21-22, and expression of rate-limiting enzymes. Studies have reported that the Warburg effect promotes drug resistance by increasing drug efflux and epigenetic alterations, mutations in drug targets, activation of survival pathways and evasion of cell death 38. In this study, we find that *C. tropicalis* promotes CRC chemoresistance by activating glycolysis pathway and producing much lactate which could down-regulate MMR functional proteins for the first time. As bacteria or fungi activate TLR receptors on the cell surface, a series of cellular changes could phosphorylate downstream AMPK. Activated AMPK can elevate glycolysis by raising the expression of PFKFB3 and exert biological effects 39. However, the specific mechanism by which *C. tropicalis* enhances glycolysis in CRC cells is not discussed in depth, so this needs our further investigation.

As a product of glycolysis, much researches have shown that lactate is no longer just a metabolic waste, but a crucial signaling molecule that participates in the regulation of metabolic pathways 20,23,25-30. GPR81, as a receptor for lactate, has now been found to couple to Gi-type protein subunit and mediate lactate-induced decrease in cAMP levels. We report that lactate down-regulates intracellular cAMP levels through GPR81 receptor in an autocrine manner, thereby reducing the nucleus entry of the PKA catalytic subunit. Hence, the transcription factor CREB in the nucleus cannot be phosphorylated, leading to decreased expression of MLH1 and promotion in the chemotherapy resistance of CRC ultimately. Accordingly, biochemical inhibition of lactate, genetic GPR81 block and activation of cAMP enhance the sensitivity of *C. tropicalis*-treated CRC cells to oxaliplatin. In this study, lactate and GPR81 are reported to be related with MMR via cAMP-PKA-CREB axis for the first time. Because glucose metabolism is a complex network with diverse intermediate products 20, whether only lactate is involved in the regulation of MMR needs further experimental confirmation.

It is of great clinical significance to clarify whether and how* C. tropicalis* participates in the chemotherapy resistance in CRC patients. This reminds us that clinical detection of *C. tropicalis* can help advance the chemotherapy effect. For patients with higher *C. tropicalis*, platinum drugs can be utilized in combination with an appropriate amount of glycolysis and LDHA inhibitors or given certain anti-fungal treatment during chemotherapy. Consequently, we should quantify levels of *C. tropicalis*, MMR functional proteins and glycolysis components especially lactate production in chemo-resistant CRC patients in the next experiments.

## Novelty and Impact

*Candida tropicalis* (*C. tropicalis*) is closely related to the occurrence and development of colorectal cancer (CRC), but whether this conditional pathogenic fungus is involved in chemoresistance remains largely unknown. Here, we find that *C. tropicalis* promotes CRC chemoresistance both *in vivo* and *in vitro*. *C. tropicalis* increases glycolysis flux and induces lactate production in colon cancer. Lactate, in turn, down-regulates the expression of MLH1 through the GPR81-cAMP-PKA-CREB axis to promote the CRC chemoresistance. These findings provide evidence that *C. tropicalis* promotes CRC chemoresistance, which may provide novel ideas for improving CRC chemotherapy effect.

## Supplementary Material

Supplementary figures.

## Figures and Tables

**Table 1:** Primer sequence

Gene	Sense (5′-3′)	Anti-sense (3′-5′)
MLH1	CAGAGCTTGGAGGGGGATA	TTTCGGGAATCATCTTCCAC
MSH2	AGGCATCCAAGGAGAATGATTG	GGAATCCACATACCCAACTCCAA
GLUT1	GGCCAAGAGTGTGCTAAAGAA	ACAGCGTTGATGCCAGACAG
HK2	GAGCCACCACTCACCCTACT	CCAGGCATTCGGCAATGTG
GPI	CAAGGACCGCTTCAACCACTT	CCAGGATGGGTGTGTTTGACC
PFKFB3	TTGGCGTCCCCACAAAAGT	AGTTGTAGGAGCTGTACTGCTT
ALDOA	ATGCCCTACCAATATCCAGCA	GCTCCCAGTGGACTCATCTG
PKM2	ATGTCGAAGCCCCATAGTGAA	TGGGTGGTGAATCAATGTCCA
LDHA	ATGGCAACTCTAAAGGATCAGC	CCAACCCCAACAACTGTAATCT
PGAM1	GTGCAGAAGAGAGCGATCCG	CGGTTAGACCCCCATAGTGC
GPR81	AATTTGGCCGTGGCTGATTTC	CCGTAAGGAACACGATGCTCC
β-actin	CATGTACGTTGCTATCCAGGC	CTCCTTAATGTCACGCACGAT
