# Supplementary material for: C. tropicalis promotes chemotherapy resistance in colon cancer through increasing lactate production to regulate the mismatch repair system
Source: Int J Biol Sci. 2021 Jul 2;17(11):2756–69. doi: 10.7150/ijbs.59262 (PMC8326116; doi:10.7150/ijbs.59262)
Supplement: Supplementary file 1 — Supplementary figures. [file ijbsv17p2756s1.pdf]

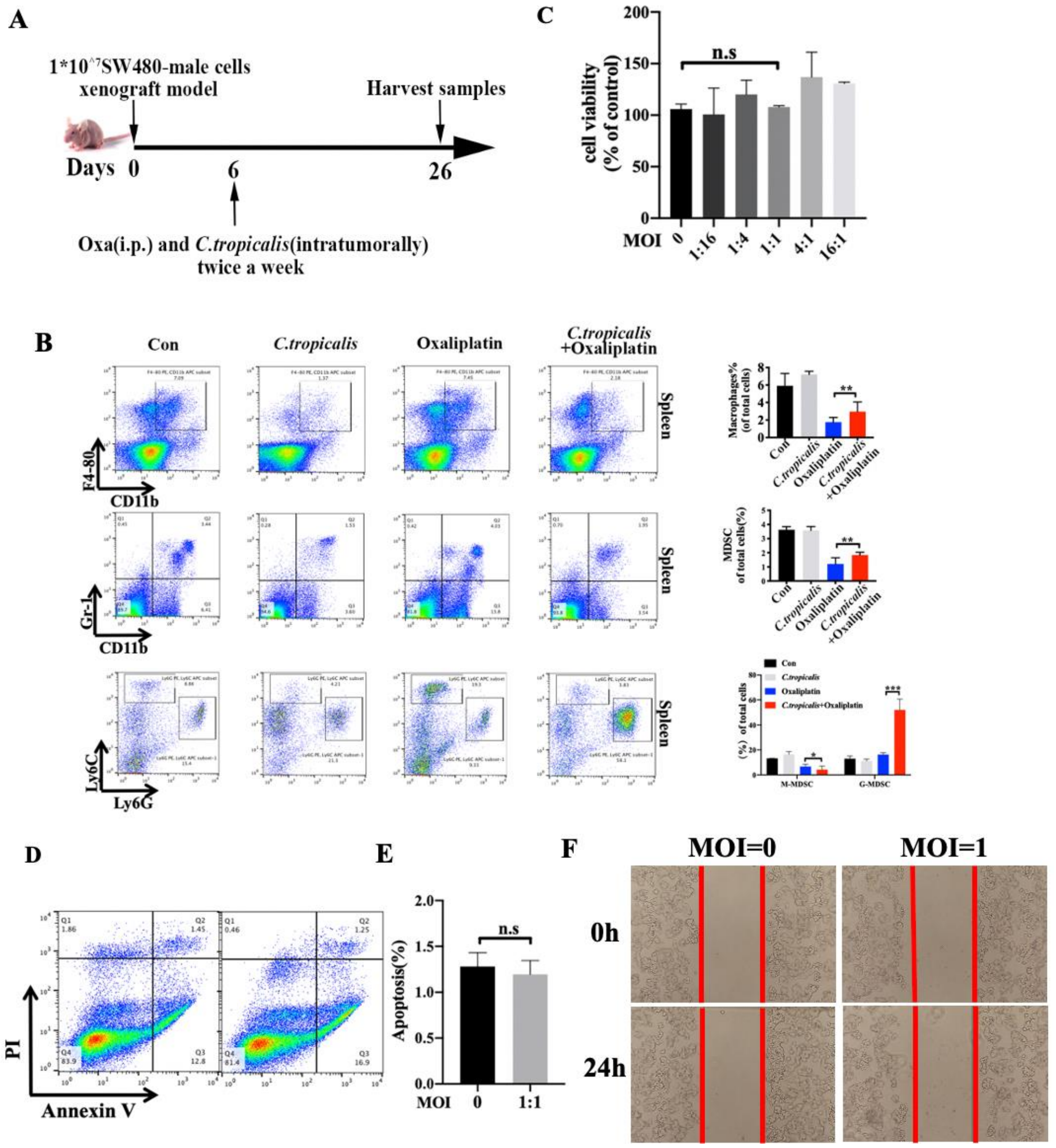

Figure S1 ***C. tropicalis*** **promotes the chemotherapy resistance of colon cancer to oxaliplatin in mouse xenograft tumor model.**

(A) Different treatment in mouse CRC xenograft model. (B) Proportions of macrophage (CD11b+F4/80+), MDSCs (CD11b+Gr-1+), G-MDSCs (CD11b+Ly6G+) and M-MDSCs (CD11b+Ly6C+) in spleen were detected by flow cytometry. (C) Cell viability was detected using CCK8 in SW480 co-cultured with different multiplicity of infection (MOI) of *C. tropicalis*. (D-E) Cell apoptosis was measured by flow cytometry in SW480 co-cultured with *C. tropicalis* (MOI = 1). (F) Wound Healing assay was performed to detect the cell migration in SW480 treated with MOI=1 *C. tropicalis*. Data with error bars are represented as mean ± SD. Each panel is a representative experiment of at least three independent biological replicates. *$p < 0.05$, **$p < 0.01$ and ***$p<0.001$ as determined by unpaired Student's t test.

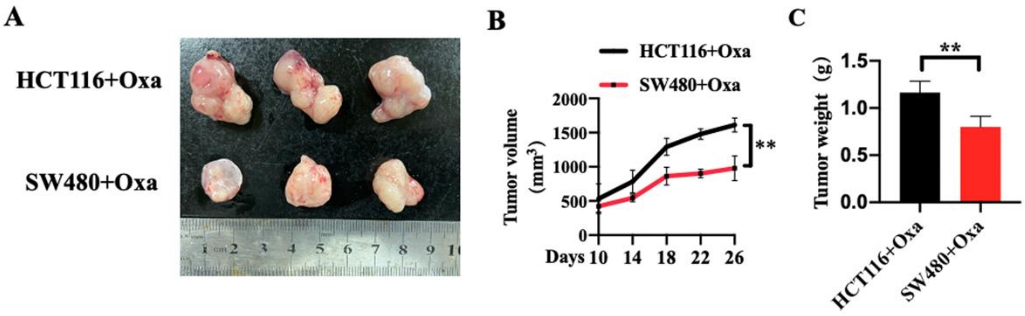

Figure S2 MMR is inhibited in *C. tropicalis*-induced CRC chemotherapy resistance.

(A) Representative image of tumors in mice injected with 5 × 10^6 HCT116-male cells or 1 × 10^7 SW480-male cells followed by oxaliplatin. (B and C) Statistical analysis of tumor volumes (B) and weights (C) in mice xenograft with HCT116 or SW480-male cells, n =3/group. Data with error bars are represented as mean ± SD. Each panel is a representative experiment of at least three independent biological replicates. *$p < 0.05$, **$p < 0.01$ as determined by unpaired Student's t test.

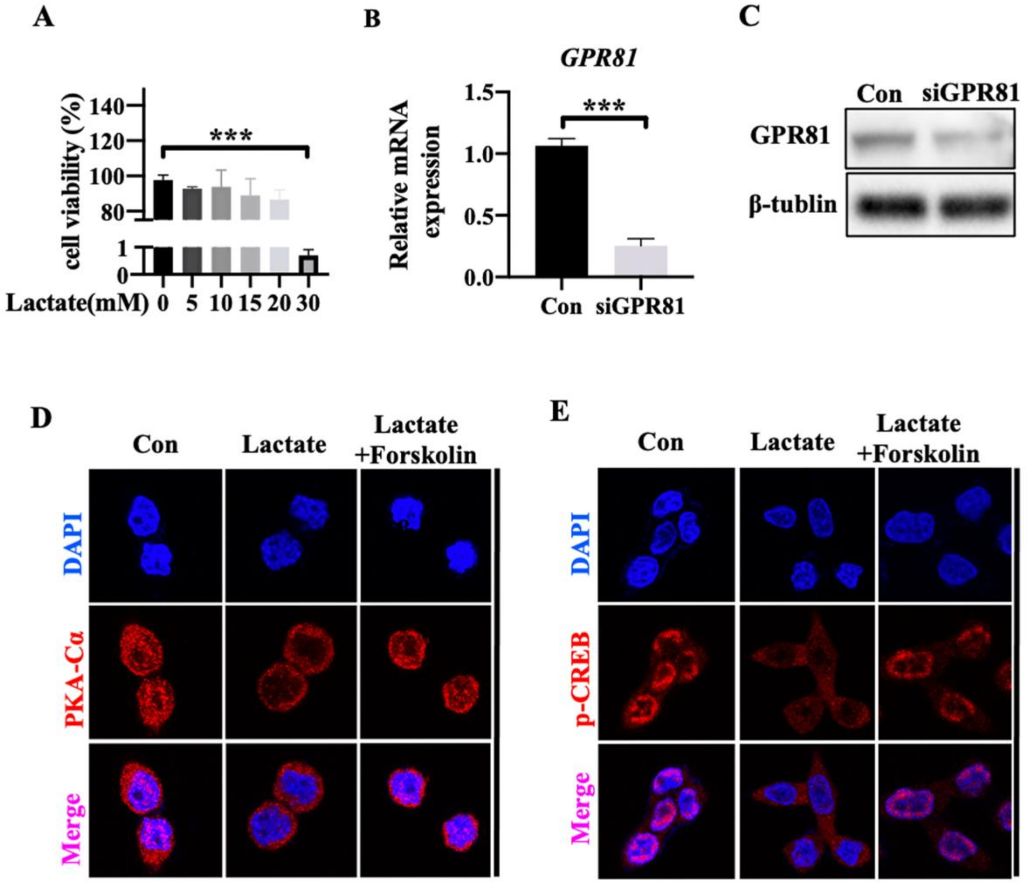

Figure S3 Lactate reduces the expression of MLH1 via GPR81-cAMP-PKA-CREB axis.

(A) Cell viability was detected using CCK8 in SW480 treated with different concentrations of lactate. (B-C) The knockdown function of GPR81 siRNA was verified by testing mRNA and protein levels of GPR81 in SW480. (D) The nucleus translocation of PKACα in the SW480 cells treated with lactate and forskolin was detected by immunofluorescence. (E) The content of intranuclear p-CREB in lactate and forskolin-treated SW480 were tested by immunofluorescence. Data with error bars are represented as mean ± SD. Each panel is a representative experiment of at least three independent biological replicates. *$p < 0.05$, **$p < 0.01$ and ***$p < 0.001$ as determined by unpaired Student's t test.
